# Editorial: Advances in genomics of plant pathogens and host-pathogen interaction

**DOI:** 10.3389/fpls.2026.1848326

**Published:** 2026-04-20

**Authors:** Abhay Kumar Pandey, Manoj Choudhary, Poulami Basak, Natasha Kashyap, Malkhan Singh Gurjar

**Affiliations:** 1 Deaprtment of Mycology & Microbiology, Tea Research Association, North Bengal Regional R & D Center, Jalpaiguri, WB, India; 2 ICAR-National Research Institute for Integrated Pest Management, New Delhi, India; 3 Division of Plant Pathology, Indian Agricultural Research Institute (ICAR), New Delhi, India

**Keywords:** agricultural crops, disease resistance, eco-friendly management, pathogenesis, pathogens

Worldwide phytopathogens are a major threat to agricultural production, causing 20 to 40% losses in crop yield annually (Ye et al., 2025). To sustain agricultural production, an efficient disease management approach must be adopted. Over the past decade, advances in genomics have significantly transformed our understanding of host–pathogen interactions by enabling high-resolution insights into the genetic and molecular basis of disease resistance (Ahmadikhah et al., 2025). On the other hand, comparative genomics has revealed the evolution of pathogen genomes, including horizontal gene transfer, and the role of transposable elements in adaptability (Pandey et al., 2023). Further, the integration of genomics with bioinformatics tools has also facilitated the discovery of resistance (R) genes in plants and molecular markers development for breeding disease-resistant crops (Chowdhury et al., 2025). In agriculture, these advances are paving the way for innovative and sustainable disease management strategies.

The Research Topic “Advances in genomics of plant pathogens and host-pathogen interaction” was aimed at next-generation sequencing technologies, comparative and population genomics, and phylogenomics that could shed light on the genomics and how pathogens intermingle with the host plant. This topic addresses research on genetic diversity, genomics, and transcriptomics of phytopathogens and their interactions with hosts provides new insights for future research. The call attracted significant interest, collecting ten accepted articles from 106 authors globally. The potential findings of these articles are discussed below.

In whole genome analysis of *Streptomyces scabiei* D6, Wei et al. identified a core set of 74 virulence-associated genes of this actinobacterium, which were closely related to the pathogenic strain *S. scabiei* LBUM848, but not with avirulent strain *S. lincolnensis* NRRL 2936. Karan et al. investigated the interaction between *Plasmopara viticola* and grapevine using genome and transcriptome approaches. The assembled genome spanning 84.09 Mb across 182 contigs, with an N50 of ~971 kb and BUSCO completeness 97%, and encodes 12,404 predicted protein-coding genes, lineage-specific expansions, and diverse transposable elements. In addition, transcriptome analysis of grapevine-infected leaves exhibited strong suppression of chloroplast- and photosynthesis-associated pathways coupled with the induction of various defense-related genes.

Win et al. characterized candidate genes associated with bacterial leaf streak (*Xanthomonas oryzae* pv. *oryzicola*) in NDCMP49, a Thai rice variety with strong resistance, using transcriptomic analysis. All varieties showed differential expression of NB-LRR proteins, receptor-like kinases, NAC transcription factors, chitinase, WRKY, and heat shock proteins at the initial stage of infection, indicating a key role for pathogen effector-triggered and pattern-triggered immunity pathways. In addition, this study also identified *Xa21*-mediated resistance to the pathogen. The researchers also studied genome-wide association on 249 rice accessions.

In transcriptome analysis of alfalfa seedling roots infected with *Sclerotium rolfsii*, Jia et al. identified 11,433 and 12,063 differentially expressed genes at control versus T24 h (24 hpi) and T4 d (4 dpi), respectively. The signal transduction and phenylpropanoid biosynthesis pathways unveiled the highest number of differentially expressed genes responsible for resistance at 24 hpi. Besides, the transcription factors, e.g. WRKY family was recognized as a key controller of several metabolic pathways linked with disease resistance. Feng et al. revealed that millet *(Setaria italica)* resistance against *Pyricularia setariae* was due to early activation of MAPK pathways. They reported downregulation of photosynthesis and ribosome-related genes in the resistant variety at the early stage of infection. Further studies revealed that this regulatory process was closely related to the MAPK signaling pathway.

In India, Kashyap et al. identified 726 miRNAs in susceptible (Agra Local) and resistant (IC566637) wheat genotypes following spot blotch (*Bipolaris sorokiniana)* infection, out of which 140 were differentially expressed and linked with the modulation of 894 genes. Further, quantitative validation of nine miRNAs using RT-PCR also supported their potential role in controlling wheat defense responses. Narvaez-Zapata et al. also studied the pathogenesis of *Fusarium verticillioides* causing maize root rot through genome sequencing and *in silico* secretome analysis. The pathogen genome size was 42.27 Mb with an N50 of 1.24 Mb and a GC content of 48.51%. Functional annotation study showed an increase in activities of various enzymes, including feruloyl esterases, pectinesterases, and glucosidases, highlighting their contribution to host cell wall degradation.

Xu et al. investigated the effects of phytopathogens on the microbial community dynamics and enzymatic activities in the potato rhizosphere using high-throughput sequencing. Urease activity was higher under *Streptomyces scabies* stress, while lower under *Phytophthora infestans* stress, whereas catalase activity reduced significantly during the full flowering and seedling phases for *Streptomyces scabies*. In addition, microbial community composition was considerably correlated with disease incidence, with specific taxa such as *Basidiomycota* and *Planctomycetes* displaying negative correlations with *Spongospora subterranea* incidence, while *Ascomycota* and *Candidatus* Dormibacteraeota were positively associated with *Phytophthora infestans*. In China, Zhao et al. identified *Thalictrum squarrosum* as an alternate host of *Puccinia triticina* and its infection phenomenon using molecular approaches. Kumar et al. also studied the genetic basis of resistance to maydis leaf blight caused by *Cochliobolus heterostrophus* and maturity-related traits in maize. There was a negative correlation between disease response and maturity-related traits, signifying that long-duration accessions are more disease resistant than short-duration accessions.

Contributions to this Research Topic emphasize the genomics and transcriptomics analysis of various agricultural plants and pathogens, the impact of microbial communities on disease progress, and the development of disease-resistant cultivars using a molecular breeding approach. The Research Topic presents all aspects that are relevant to genomics of plant pathogens and host-pathogen interaction. Despite these remarkable achievements, several challenges remain. It is still difficult to characterize genes identified through genomic studies, and interdisciplinary collaboration is required to translate genomic data into field-level applications. Further, it is imperative that genomic databases are continually monitored and updated in light of the rapid evolution of pathogens. Ultimately, genomics has revolutionized plant pathology by providing a comprehensive framework for understanding host-pathogen interactions including disease resistance at the molecular level. In addition, early disease management and surveillance are also being improved by advances in pathogen diagnostics, including rapid molecular detection techniques. To develop resilient agricultural systems capable of withstanding emerging plant diseases, genomics must be integrated with breeding, biotechnology, and ecological approaches.
